# Unraveling the Role of RNA Mediated Toxicity of *C9orf72* Repeats in C9-FTD/ALS

**DOI:** 10.3389/fnins.2017.00711

**Published:** 2017-12-15

**Authors:** Vijay Kumar, Gulam M. Hasan, Md. Imtaiyaz Hassan

**Affiliations:** ^1^Centre for Interdisciplinary Research in Basic Sciences, Jamia Millia Islamia, Jamia Nagar, India; ^2^Department of Biochemistry, College of Medicine, Prince Sattam Bin Abdulaziz University, Al-Kharj, Saudi Arabia

**Keywords:** *C9orf72*, C9-FTD/ALS, hexanucleotide repeat expansions, RNA-binding proteins, transcriptome, pathomechanisms

## Abstract

The most frequent genetic cause of amyotrophic lateral sclerosis (ALS) and frontotemporal dementia (FTD) is intronic hexanucleotide (G4C2) repeat expansions (HRE) in the *C9orf72* gene. The non-exclusive pathogenic mechanisms by which C9orf72 repeat expansions contribute to these neurological disorders include loss of C9orf72 function and gain-of-function determined by toxic RNA molecules and dipeptides repeats protein toxicity. The expanded repeats are transcribed bidirectionally and forms RNA foci in the central nervous system, and sequester key RNA-binding proteins (RBPs) leading to impairment in RNA processing events. Many studies report widespread transcriptome changes in ALS carrying a C9orf72 repeat expansion. Here we review the contribution of RNA foci interaction with RBPs as well as transcriptome changes involved in the pathogenesis of C9orf72- associated FTD/ALS. These informations are essential to elucidate the pathology and therapeutic intervention of ALS and/or FTD.

## Introduction

Amyotrophic lateral sclerosis (ALS) is a devastating adult-onset neurodegenerative disorder which involves upper and lower motor neuronal loss that finally leads to paralysis and death. Frontotemporal dementia (FTD) is a complex dementia disturbing language, cognitive and behavioral skills. Both are fatal within 3–5 years of symptoms onset (Ratnavalli et al., 2002; Pasinelli and Brown, 2006). In 2011, a common basis for ALS and FTD was identified as a hexanucleotide repeat expansion of the G4C2 (HRE) in the non-coding region of the chromosome 9 open reading frame 72 (*C9orf72*) gene, referred as C9-FTD/ALS (DeJesus-Hernandez et al., 2011; Renton et al., 2011). Further studies reveal the same mutations making the *C9orf72* repeat expansion the most common known genetic cause of C9-FTD/ALS to date in an increasing number of patients (Gijselinck et al., 2012; Majounie et al., 2012; Ling et al., 2013). The size of the repeat in ALS and FTD cases ranges between 700 and 1,600 as compared to 2–23 in controls (DeJesus-Hernandez et al., 2011).

C9orf72 is a highly conserved protein throughout the evolution, expressed mostly within the central nervous system (CNS), and is indistinctly related to the differentially expressed in normal and neoplastic cells (DENN) family of GDP–GTP change factors activating Rab GTPases (Levine et al., 2013; Suzuki et al., 2013). The genetic mutations of *C9orf72* show autosomal dominant inheritance (Hosler et al., 2000). Patients with C9-FTD/ALS are clinically characterized by the earlier disease onset with bulbar involvement, cognitive and behavioral impairment with psychosis and Parkinsonism in many cases (Cruts et al., 2013; Cooper-Knock et al., 2014a).

**Figure 1:**
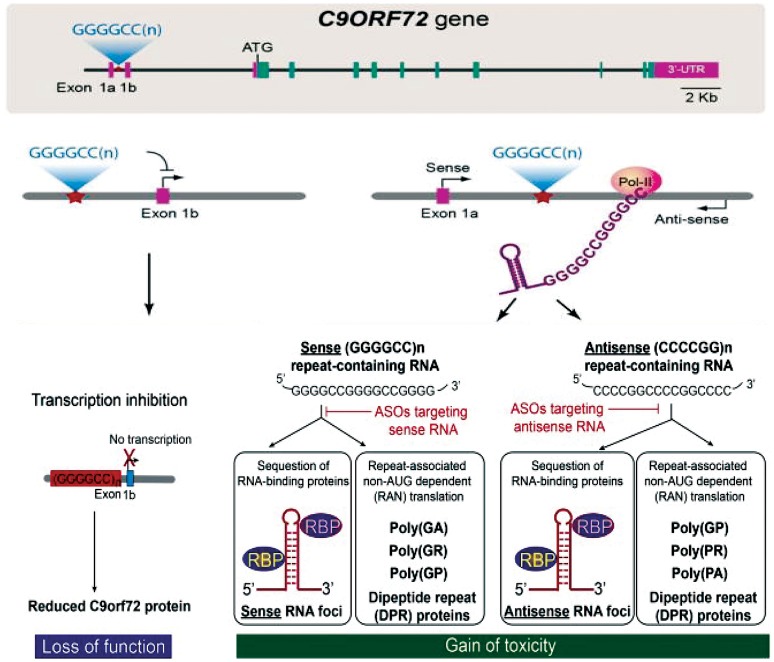
C9orf72 repeat associated disease mechanisms and therapy development. Three major hypothesis explaining the pathomechanisms are loss of protein function and/or gain of toxicity from repeat containing RNAs and production of dipeptide repeat proteins through repeat-associated non-AUG-dependent (RAN) translation. Figure adapted from Jiang and Cleveland (2016).

There are currently three mechanisms that have been suggested to explain how these repeat expansions cause disease (Figure 1). First, the presence of repeat expansion cause down regulation of *C9orf72* gene expression leading to the loss-of-function (DeJesus-Hernandez et al., 2011). Second, RNA-mediated gain-of-function by sequestration of essential RNA-binding proteins (RBPs) into intranuclear RNA foci (Gendron et al., 2014). Third, production of dipeptide repeats (DPR) proteins through unconventional non-AUG-dependent (RAN) translation of repeat containing RNAs (Gendron et al., 2013). These three mechanisms are not completely mutually exclusive, but overall define the major disease mechanism and will be essential for the therapeutic interventions. We refer the readers to references (Rohrer et al., 2015; Gitler and Tsuiji, 2016; Todd and Petrucelli, 2016) to get additional insights into the mechanisms of C9orf72-mediated neurotoxicity in detail.

## Loss-of-function

C9orf72 plays key role in membrane trafficking as a Rab guanine exchange factor (Levine et al., 2013) and Rab GTPase-dependent autophagy regulation (Sellier et al., 2016; Webster et al., 2016). C9orf72 HRE decreased the expression of C9orf72 variant 2 transcript levels (DeJesus-Hernandez et al., 2011; Gijselinck et al., 2012). This decrease in expression could trigger disease by haploinsufficiency. Other studies have demonstrated that the expanded repeats also interfere with transcription or splicing of other two variants (Mori et al., 2013b; Highley et al., 2014). Further studies have reported reduction of C9orf72 expression in induced pluripotent stem cell (iPSC) neurons and brain from C9-FTD/ALS patients (Almeida et al., 2013; Belzil et al., 2013; Donnelly et al., 2013; Waite et al., 2014). One hypothesis explaining the haploinsufficiency is the hypermethylation of CpG islands at 5' end of the G4C2 repeat observed in ALS and FTD carriers (Xi et al., 2013). In addition, trimethylation of histones H3 and H4 lysine residues that binds tightly to G4C2 repeats in brain tissue provides another epigenetic mechanism involved in reducing gene expression (Belzil et al., 2013). Moreover, the G4C2 repeat expansion could also disrupt the *C9orf72* promoter activity (Gijselinck et al., 2016). Alternatively, the expanded G4C2 repeat can form G-quadruplex and R-loop structures (Reddy et al., 2013; Kumar et al., 2016) that could result in abortive transcription of C9orf72 (Haeusler et al., 2014).

However, loss of C9orf72 function alone does not seem to be sufficient to cause ALS/FTD, as conditional knockdown of C9orf72 mice does not induce motor neuronal loss (Jiang et al., 2016; O'rourke et al., 2016). On the other hand, gain of toxic function through RNA foci or DPR proteins in different model systems produces motor defects and ALS/FTD-like symptoms (Chew et al., 2015; Jiang et al., 2016; Liu et al., 2016).

## RNA-gain-of-function toxicity

Nearly all of the non-coding repeat expansion diseases are characterized by the presence of cellular aggregates of mutant RNA termed as RNA foci (Greco et al., 2002; La Spada and Taylor, 2010; DeJesus-Hernandez et al., 2011). These are dynamic structures characterized by random association and dissociation of RBPs with mutant repeat RNA (Wojciechowska and Krzyzosiak, 2011). C9orf72 HRE could cause disease by sequestering the key RBPs and form intranuclear RNA foci detected in brain and spinal cord of C9-FTD/ALS patients (DeJesus-Hernandez et al., 2011). Foci composed of the sense strand of the repeat RNA are detected in nuclei and rarely in the cytoplasm (Lagier-Tourenne et al., 2013; Mizielinska et al., 2013; Zu et al., 2013). Adding to the complexity of the pathomechanisms, it was subsequently shown that the antisense repeat RNA resulting from the bidirectional transcription of HRE, accumulated in distinct RNA foci in frontal cortex, spinal cord, and cerebellum of C9-FTD/ALS patients (Gendron et al., 2013). Sense and antisense foci are generally found in separate cells, but can be present within the same cell or nucleus (Mizielinska et al., 2013; Zu et al., 2013). These RNA foci are significantly enriched with RBPs involved in various RNA metabolism events like splicing, translation and transport. Recently many researchers have focused on to identify RBPs sequestered by RNA foci and to determine how their loss of function contributes to disease. We have discussed some of the well known RBPs involved in C9-FTD/ALS in the subsequent section of the review.

## DPR protein toxicity

The third potential pathomechanisms has been based on the findings that the bidirectionally transcribed repeat expansions can escape the nucleus, associate with ribosomal complex and being translated by RAN translation into five different aggregating DPR proteins in C9-FTD/ALS (Ash et al., 2013; Mori et al., 2013b; Zu et al., 2013; Zhang et al., 2014). These DPRs are: poly-(Gly-Ala) (GA) and poly-(Gly-Arg) (GR) from sense transcripts, poly-(Pro-Arg) (PR) and poly-(Pro-Ala) (PA) from antisense transcripts, and poly-(Gly-Pro) (GP) from both the sense and antisense transcripts.

A large number of studies have provided evidences that these DPRs are involved in the pathology of C9-FTD/ALS (Kwon et al., 2014; May et al., 2014; Mizielinska et al., 2014; Wen et al., 2014; Yang et al., 2015; Zhang et al., 2016). For instance, GA proteins induced loss of proteasome activity, endoplasmic stress and neurotoxicity in the absence of RNA foci in primary neurons (Zhang et al., 2014). GA proteins have also been demonstrated to form toxic amyloids and may show prion-like activity (Chang et al., 2016). Many recent studies have reported the involvement of arginine-rich DPRs (GR and PR) in the formation of membrane-less organelles and liquid-liquid phase separation (Lee et al., 2016; Lin et al., 2016). Through proteomic analysis, the binding partners of GR and PR have been identified that are enriched in proteins containing low-complexity domains (LCDs) and are often present in the nucleolus (Lee et al., 2016; Lin et al., 2016). The alterations of liquid-like properties by GR and PR DPRs illustrate a potential mechanism by which DPRs induce splicing defects (Freibaum and Taylor, 2017). Also, these repeats contribute to the toxicity and lethality in Drosophila model (Mizielinska et al., 2014; Wen et al., 2014). Moreover, Tao et al. (2015) showed that the GR and PR DPRs induce nucleolar stress and impaired stress granule formation. Now, accumulating evidences support that aberrant stress granule assembly during cellular stress is an important pathomechanisms in C9-FTD/ALS (Buchan, 2014; Kwon et al., 2014; Wen et al., 2014; Tao et al., 2015).

Interestingly, a recent study identified that the DPR-aggregates contain Drosha protein, a key player in miRNA biogenesis (Porta et al., 2015). The Drosha protein forms neuronal cytoplasmic inclusions colocalized with p62 and ubiquilin-2 in the frontal cortex and cerebellum of C9-FTD/ALS patients. The mislocalization of the Drosha protein thus indicates the disruption RNA/miRNA processing in C9-FTD/ALS.

### RBPs sequestration in C9-FTD/ALS

Recent advances in microarray and next-generation sequencing technologies enable us to study the global analysis of genome, transcriptome, proteome, and metabolome, collectively termed as “omics.” These omics studies help us to understand the genome-wide molecular basis of diseases and to identify disease associated molecular biomarkers. Sequestration of RBPs and the presence of nuclear RNA foci indicate that the mutation may alter the cellular transcriptome, which could be targeted for therapeutic intervention.

**Figure 2:**
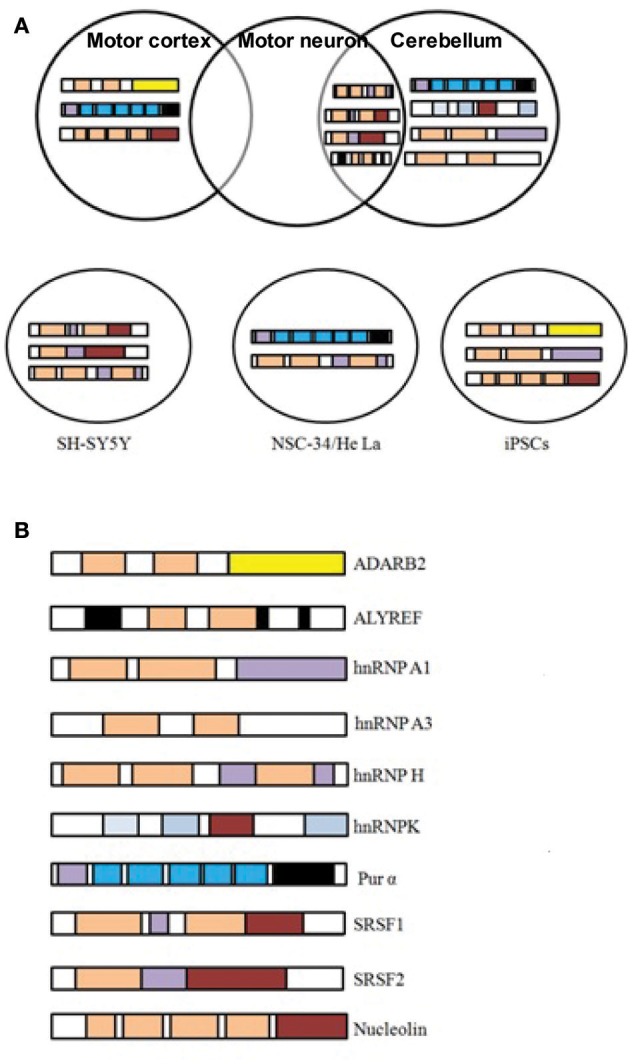
*C9orf72* repeat RNA-binding proteins. **(A)** RBPs shown to co-localize *in vivo* with *C9orf72* hexanucleotide repeat RNA foci of patient tissues or in cellular models. Figures were made from the data in references shown in Table 1. **(B)** Modular structure of selected RBPs.

Based on the fact that RNA foci in C9-FTD/ALS sequester RBPs, many studies undertake the challenge to identify proteins that bind and co-localize with RNA foci. A large number of such proteins were recently identified (Haeusler et al., 2014). The biggest group among them is the heterogeneous nuclear ribonucleoprotein group (hnRNPs). hnRNPA1 (Mori et al., 2013a; Sareen et al., 2013), hnRNPA2/B1(Almeida et al., 2013; Mori et al., 2013a), hnRNPA3 (Mori et al., 2013a), hnRNPH (Lee et al., 2013), hnRNPF (Haeusler et al., 2014), hnRNPK (Haeusler et al., 2014), hnRNPL (Mori et al., 2013a), hnRNPU (Haeusler et al., 2014), and others (Vatovec et al., 2014) were reported by different groups to interact with G4C2 RNA repeats. For many of these RBPs sequestration and co-localization studies, iPSC-derived neurons, cultured cell models, mouse primary neurons and patients tissues have been used to model C9-FTD/ALS. Eventually, different studies have identified many RBPs whose functions were disturbed in C9-FTD/ALS (Table 1). Here, we summarize the findings of some of the better-characterized RBPs. Figure 2 shows co-localization of RBPs with RNA foci in patient's tissues and in cellular models.

**Table 1:** Summary of studies describing *C9orf72* hexanucleotide repeat RNA-binding proteins.

**S.N**.	**System**	**Number of RBPs**	**References**
1	HEK293T	288	Haeusler et al., 2014
2	HEK293 cells	235	Mori et al., 2013a
3	Mouse spinal cord	236	Xu et al., 2013
4	SH-SY5Y cell Human cerebellum	69 43	Cooper-Knock et al., 2014b
5	Mouse brain	30	Almeida et al., 2013
6	Proteome array	19	Donnelly et al., 2013
7	SH-SY5Y cells	3	Lee et al., 2013
8	iPSCs from skin fibroblasts	2	Sareen et al., 2013
9	NSC34 and HeLa cells	14	Rossi et al., 2015

#### HNRNPA3

Mori et al. (2013a) performed an RNA pull down assay using *in vitro*-transcribed (G4C2)23 RNA incubated with nuclear extracts from human embryonic kidney (HEK) 293 cells. Subsequently, they identified 20 top candidate RBPs including hnRNPs, splicing factors and mRNA-binding proteins. A subset of these proteins was further investigated by immunohistochemical analysis on patient hippocampus. This analysis showed hnRNPA3 as a component of the neuronal cytoplasmic and nuclear inclusions. These results suggest that binding of the hnRNPA3 to G4C2 repeats initiate aberrant export of *C9orf72* pre-mRNA to the cytosol for RAN translation or its subsequent degradation. However, the co-localization between hnRNPA3 and nuclear RNA foci in patient cerebellum (Lee et al., 2013) or iPSC-derived motor neurons (Sareen et al., 2013) has not been validated.

#### HNRNPH

Close co-localization was also demonstrated between neuronal G4C2 RNA foci and hnRNPH protein in ALS and FTD brain tissues. Lee et al. (2013) has shown that the binding of hnRNPH enhances the aggregation of G4C2 RNA and forms large RNA foci. Using neuronal cell model SH-SY5Y, the authors performed immunofluorescent studies of 30 different RBPs coupled with RNA fluorescence *in situ* hybridization (FISH). They found that only hnRNPH, serine-arginine-rich splicing factor 1 (SRSF1), and serine-arginine-rich splicing factor 2 (SRSF2) co-localized with intranuclear G4C2-positive RNA foci. Further, they showed that only hnRNPH binds the G4C2 repeats in co-immunoprecipitation assay and co-localizes with 70% of RNA foci detected in patient cerebellum. The authors went further to show that hnRNPH sequestration in RNA foci decreases the alternative splicing of TARBP2, a known RNA target of hnRNPH, implying the loss of hnRNPH function. It has also been demonstrated that hnRNP H co-localizes with RNA foci in NSC34 and HeLa cells (Rossi et al., 2015).

#### PUR-α

Pur-α plays an essential role in FXTAS and bind to CGG repeats, along with hnRNPA2/B1 (Jin et al., 2007). Pur α, Pur β, and Pur γ were the most interacting RBPs in mouse spinal cord lysates (Xu et al., 2013). Pur α binds G4C2 repeats in a dose dependent manner and modulates the toxicity of the G4C2 RNA foci in mammal and Drosophila models. However, co-localization of Pur-α and RNA foci was confirmed by some groups (Sareen et al., 2013; Xu et al., 2013; Rossi et al., 2015), but not by other groups (Donnelly et al., 2013; Lee et al., 2013). It has been shown that Pur-α rescued repeat induced cell death and knock-down of Pur-α in Neuro2a cells resulted in increased cytotoxicity (Xu et al., 2013). Rossi et al. (2015) recently showed that Pur-α form nuclear and cytoplasmic puncta in NSC34 and HeLa cells expressing (G4C2)31 repeat. These puncta co-localized occasionally with RNA foci in the nucleus, but in the cytoplasm it is co-localized with stress-granule marker, demonstrating the mislocalization of RBPs to stress granules in C9-FTD/ALS.

#### ADARB2

Donnelly et al. (2013) employed a human proteome array and showed that adenosine deaminase RNA-specific, B2 (ADARB2) binds with (G4C2)6.5 repeats. They also showed that ADARB2 co-localizes with nuclear RNA foci in *C9orf72* iPSC-derived motor neurons (iPSNs) as well as in the motor cortex of C9-FTD/ALS patients. Knock-down of ADARB2 drastically reduces the formation of RNA foci, suggesting the functional role of ADARB2 in *C9orf72*-mediated RNA toxicity.

#### Nucleolin

Haeusler et al. (2014) also identified a number of RBPs that binds different RNA repeats in HEK-293T cells through SILAC-based screen. Follow-up analysis using RNA pull down and western blot showed that nucleolin (NCL) and hnRNPU specifically binds the RNA G-quadruplex motif, hnRNPF and ribonucleoprotein (RPL7) bind both the G-quadruplex and hairpin structures, while hnRNPK binds preferentially to the antisense repeat. The authors further showed that Nucleolin and nucleophosmin has a more diffuse pattern in B-lymphocytes, fibroblasts, and iPSNs of C9-FTD/ALS patient cells. Some neurons revealed disrupted pattern of nucleolin in the motor cortex of C9-FTD/ALS patients also. Infrequent co-localization of nucleolin to sense RNA foci in C9-FTD/ALS cases suggest an involvement of the repeat RNA in the disturbance of nucleolar function (Haeusler et al., 2014; Cooper-Knock et al., 2015b).

#### SRSF2 and ALYREF

Cooper-Knock et al. (2014b) carried out RNA pull down assay using SH-SY5Y cells and human cerebellum and identified proteins involved in mRNA nuclear export, translation and splicing. Follow-up analysis of C9-FTD/ALS patients presented that RNA foci co-localizes with SRSF2, hnRNPH/F, and ALYREF in cerebellar granule cells as well as in motor neurons, while hnRNPA1 co-localizes only with cerebellar granule cells. Moreover, hnRNPA1 also co-localizes with RNA foci in C9-FTD/ALS patient-derived iPSNs (Sareen et al., 2013), whereas SRSF1 and SRSF2 co-localizes with cerebellar RNA foci, but is relatively occasional (Lee et al., 2013). Recently, Hautbergue et al. (2017) showed that SRSF1 binds to C9orf72 repeats and promotes nuclear export of these pathological repeats. Knockdown of SRSF1 is thus protective in multiple C9-FTD/ALS models.

Despite, several studies identify and discuss repeat- binding RBPs, yet there is disparity among each study, and the results were not always replicated in other animal models or patient tissues. This discrepancy could be due to the variations in methodologies used. The different cellular models, mouse lysates or patient tissues used for these studies might express a different group of proteins than that observed actually in human CNS and therefore could result in strong false positive hits. Moreover, the lengths of the repeats studied are not similar to what was observed in patients. All of these factors could contribute in biasness of the results. Moreover, the role of different RBPs to the pathologies of the disease is also not very clear. It is still unclear whether the disruption of RBPs function is because of their binding or co-localization with RNA foci. Detailed follow-up studies are needed to show that these events affect RBPs endogenous functions and contribute to pathology.

### Transcriptional changes and network analysis of C9-FTD/ALS pathology

**Figure 3:**
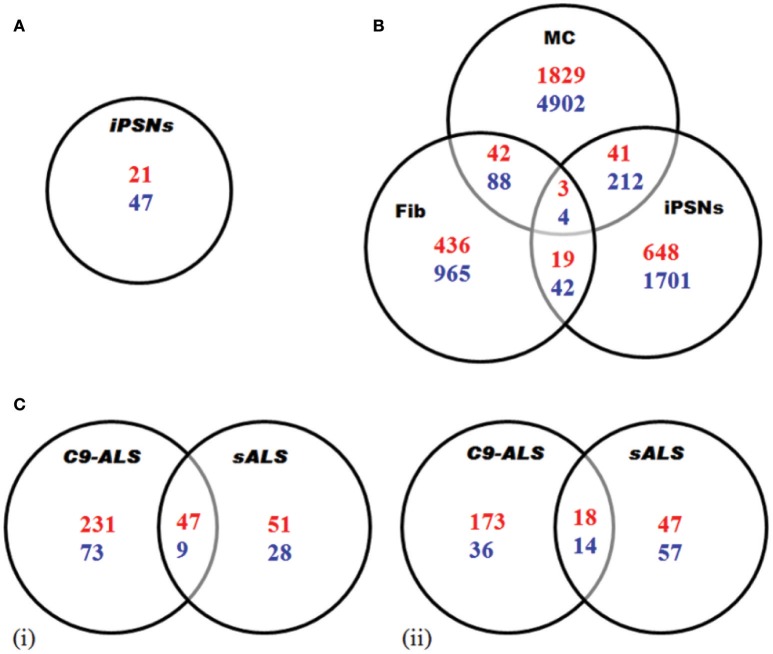
Transcriptome analysis of C9-ALS. Here, up-regulated (blue) and down-regulated (red) genes are represented. **(A)** Gene expression analysis via RNA-seq in iPSNs from C9-ALS patients. A total of 68 genes showed differential expression. **(B)** Gene expression study via microarray analysis in *C9orf72* fibroblasts (Fib), iPSC-derived motor neurons (iPSNs) and motor cortex (MC). **(C)** Differential regulation of gene expression analysis in C9-ALS and sALS cases. Venn diagrams representing up-regulated and down-regulated genes in cerebellum (i) and frontal cortex (ii). Figures were made from the data in references shown in Table 2.

There is utmost requirement to underpin the mechanisms of pathogenesis in C9-FTD/ALS. *C9orf72* repeat expansion exerts direct effect on the transcriptome via the formation of RNA foci (DeJesus-Hernandez et al., 2011; Cooper-Knock et al., 2014b). Thus, transcriptome changes associated with the disease may lie under the disease pathogenesis and represent a suitable therapeutic target. A number of studies have been done to identify transcriptional changes using different model systems or patient tissues. We tried to briefly summarize these studies here and depicted the results in Figure 3 and Table 2.

**Table 2:** Gene ontology (GO) terms based on analysis of up- or down-regulated genes in C9-ALS cases.

	**Up-regulated**	**Down-regulated**	**References**
	Extracellular matrix	Neuron differentiation	Sareen et al., 2013
	Cell adhesion	Cell-cell signaling	
	Cell-cell signaling	Synapse	
	Synaptic transmission		
	Neurological process		
Motor neurons	RNA splicing	Cholesterol biosynthesis	Cooper-Knock et al., 2015a
	Erythrocyte homeostasis	Regulation of glucose metabolism	
	Male sex differentiation	Regulation of nuclear division	
Lymphoblastod cell lines	RNA splicing	Inflammatory response	
	Protein catabolic process	Regulation of action potential in neuron	
	Synaptic transmission	Striated muscle tissue development	
	Positive regulation of apoptosis		
Cerebellum	Pattern specification process	G-protein coupled receptor protein signaling pathway	Prudencio et al., 2015
	Skeletal system development	Cognition	
	Embryonic morphogenesis	Regulation of nucleotide biosynthetic process	
	Response to unfolded protein	Immune response	
	Inflammatory response	Regulation of nucleotide metabolic process	
Frontal Cortex	Inflammatory response	Gas transport	
	Response to wounding	Oxygen transport	
	Defence response	Haemoglobin metabolic process	
	Response to unfolded protein		
	Digestion		

Using iPSNs, Sareen et al. (2013) utilized RNA-seq to identify dissimilar transcriptional profiles for C9-FTD/ALS when compared to controls. The numbers of up-regulated genes were twice as compared to down-regulated genes. Functional pathway analysis suggested that the upregulated genes are involved in cell adhesion, synaptic transmission and neural differentiation (Figure 3A).

Similarly, the other study by Donnelly et al. (2013) identifies unique gene expressions alteration in C9-FTD/ALS fibroblast, iPSNs, and motor cortex when compared to healthy controls as well as SOD1^mut^ fibroblasts and iPSN lines. They found that large numbers of genes are down-regulated. They also demonstrated the overlap between the expression changes seen in iPSNs and C9-FTD/ALS motor cortex, suggesting that these iPSNs could recapitulate at least some disease-specific gene alterations (Figure 3B). Lagier-Tourenne et al. (2013) studied the RNA expression changes in fibroblasts from C9-FTD/ALS and sALS patients, and reported that RNA expression profile is unique for each group. Cooper-Knock et al. (2015a) analyzed transcriptome in lymphoblastoid cells and motor neurons of C9-FTD/ALS cases. They reported the up-regulation of “RNA splicing” genes in motor neurons and lymphoblastoid cell lines of patients with C9-FTD/ALS. Up-regulation of these genes is consistent with an effort to compensate these questration of these proteins by the RNA foci. Moreover, the authors went further to show differential expression in RNA splicing factors and lower splicing in C9-FTD/ALS patients than in controls. Prudencio et al. (2015) also reported large transcriptome alterations in the frontal cortex and cerebellum of C9-FTD/ALS and sALS cases when compared to healthy controls. They showed that C9-FTD/ALS transcriptome was altered more compared to sALS (Figure 3C). A huge disruption of alternative splicing events in C9-FTD/ALS than sALS cases has been observed, along with disease-specific changes in gene expression. Gene ontology (GO) analyses showed that unfolded protein response (UPR) and the protein transport machinery are mainly affected in C9-FTD/ALS whereas, cytoskeleton organization, defense response and synaptic transmission pathways are mainly affected in sALS.

We now know that *C9orf72* repeats sequester several RBPs and form nuclear RNA foci thus alter the cellular transcriptome by aberrantly affecting a number of biological pathways. Table 2 summarizes the differentially expressed networks in C9-FTD/ALS.

To identify biologically relevant pathways in C9-FTD/ALS pathology, Satoh et al. (2014) used different pathway analysis tools including Kyoto Encyclopedia of Genes and Genomes (KEGG:www.kegg.jp), Ingenuity Pathways Analysis (IPA:www.ingenuity.com), and KeyMolnet (www.km-data.jp/keymolnet) to study molecular networks engaged in C9-FTD/ALS by utilizing three different *C9orf72* omics datasets. These data sets were: (i) proteome of *C9orf72* HRE RBP, which provides the most important biochemical information of C9-FTD/ALS (Cooper-Knock et al., 2014b; Haeusler et al., 2014); (ii) transcriptome of iPSNs of patients with C9-ALS (Sareen et al., 2013); and (iii) transcriptome of motor neurons of C9-FTD/ALS patients acting as the most clinically appropriate *in vivo* source (Highley et al., 2014).

The results of this study reveal *C9orf72* HREs involvement in the ribosome function, spliceosome, and post-transcriptional modification of RNA. Essentially, the proteome is enriched of RBPs having RNA-recognition motifs and prion like domains. Similarly, a network analysis of differentially expressed genes in iPSNs of patients with C9-ALS shows that the majority of genes identified were under expressed, namely the genes encoding for extracellular matrix proteins and matrix metalloproteinases. Moreover, the authors did not observed any significant differences in splicing patterns of C9-ALS patients and controls. In addition, the authors also reported that the post-transcriptional RNA processing, cytoskeletal dynamics and intracellular molecular transport have been affected in C9-ALS patients.

### Therapy for RNA-gain-of toxicity

Overall, the potential pathomechanisms associated with C9-FTD/ALS may represent possible therapeutic interventions. Considering that the RNA foci and/or DPRs are important components of the disease, selectively inhibition of their transcription and translation might reduce the disease burden. Significant advances have been achieved in this direction including utilization of antisense oligonucleotides (Figure 1) and RNA interferences (Donnelly et al., 2013; Lagier-Tourenne et al., 2013; Sareen et al., 2013; Mis et al., 2017). In addition, small molecules targeting the secondary structure of repeat RNA, limiting the accumulation of RNA foci and toxic DPRs (Su et al., 2014). Moreover, gene therapy can be used to silence the toxic RNA/protein and also reduces the haploinsufficiency (Deng et al., 2014; Donnelly et al., 2014; Xiao-Jie et al., 2015). Given the plethora of pathomechanisms of C9-FTD/ALS, we propose that multi-targeted therapeutic approaches should be needed to treat patients with C9-FTD/ALS.

## Concluding remarks and future perspective

There are intense discussions in the C9orf72 field on the pathomechanisms and as reviewed here, there is possibility that a combination of multiple mechanisms may be involved in causing the disease. For example, the reduced level of functional C9orf72 could increase neuronal susceptibility to RNA foci or DPRs. Overall, disturbance of normal RNA processing is one of the key pathological events of C9-FTD/ALS. RNA metabolism and RNA-binding proteins are recurrent themes in neurodegeneration and is crucial for neuronal survival. The *C9orf72* repeat expansion contributes to neurodegeneration through the formation of nuclear RNA foci that sequester, and cause functional loss of key RBPs results in the defects in RNA processing and gene expression. Several RBPs co-localize with RNA foci in C9-FTD/ALS brain tissues and/or neurons differentiated from iPSCs. Furthermore, sequestration of RBPs may change the cellular transcriptome which could offer a better therapeutic intervention. Importantly, disease models such as cultured cells or motor neurons does o not cover the different brain regions affected in C9-FTD/ALS, thus may not reflect disease processes accurately. Future studies seeking the cause of pathomechanisms in disease-relevant models and patient tissue will be crucial to understand disease pathogenesis and prevention. At present, it seems that we have just begun to understand the disease pathogenesis. As our understanding of pathomechanisms underlying C9-FTD/ALS pathogenesis grows, so also will the possibility of developing effective therapeutic strategies.

## Author contributions

VK designed the topic; VK, GH, and MH wrote the paper.

### Conflict of interest statement

The authors declare that the research was conducted in the absence of any commercial or financial relationships that could be construed as a potential conflict of interest.
